# The diagnostic of PAX1 gene methylation in cervical lesions and its role in the triage of non-16/18 HR-HPV positive

**DOI:** 10.3389/fimmu.2025.1634297

**Published:** 2025-07-24

**Authors:** Meng-Meng Chen, Bing-Qiang Zhang, Yan-Song Luan, Guo-Long Sun, Yun-Yuan Zhang, Xiao-Yan Zhou, Xi-Feng Zhang, Feng-Chun Gao, Jun-Mei Yu

**Affiliations:** ^1^ Qingdao Restore Medical Laboratory Co., Ltd., Qingdao, Shandong, China; ^2^ Key Laboratory of Cancer and Immune Cells of Qingdao, Qingdao, China; ^3^ Department of Clinical Laboratory, The Affiliated Hospital of Qingdao University, Qingdao, Shandong, China; ^4^ College of Veterinary Medicine, Qingdao Agricultural University, Qingdao, China; ^5^ Department of Obstetrics, Jinan Materlity and Child Care Hospital Affiliated to Shandong First Medical University, Jinan, China

**Keywords:** cervical cancer, methylation, *PAX1*, HR-HPV, TCT

## Abstract

**Objective:**

This study aims to systematically evaluate the application value of *PAX1* gene methylation detection in cervical lesion screening and its potential advantages in the triage of non-16/18 high-risk human papillomavirus (HR-HPV) positive patients.

**Materials and methods:**

This study enrolled 1,619 HPV-positive female patients who visited the Affiliated Hospital of Qingdao University from December 2023 to March 2025, with 989 patients ultimately meeting the inclusion criteria. All subjects underwent HPV-DNA testing, cytological examination, colposcopy, and *PAX1* gene methylation detection, with results analyzed in conjunction with histopathological evaluations. HPV-DNA detection was performed using fluorescence quantitative PCR methodology capable of identifying 17 high-risk HPV genotypes. Cytological examination results were classified according to the International Society of Cytology standards. *PAX1* gene methylation detection employed fluorescence quantitative PCR technology with ACTB as the internal reference gene, determining methylation levels through calculation of ΔCT values. Statistical analyses included ROC curve assessment of diagnostic performance, with intergroup comparisons conducted using one-way analysis of variance and Pearson’s chi-squared test.

**Results:**

The results demonstrated that PAX1 gene methylation detection showed significantly better diagnostic performance compared to cytological examination for the detection of CIN2+ and CIN3+ lesions. The AUC values for *PAX1* gene methylation detection in diagnosing CIN2+and CIN3+ were 0.934 (95% confidence interval [CI]: 0.916–0.948) with sensitivity of 93.49% and specificity of 93.24%and0.875 (95% confidence interval [CI]: 0.853–0.895)with sensitivity of 95.31% and specificity of 79.77%. Among non-16/18 HR-HPV(in women positive for high-risk HPV types other than 16/18) positive patients, *PAX1* gene methylation detection demonstrated higher sensitivity and specificity than cytological examination, enabling more accurate identification of patients requiring further intervention and reducing unnecessary colposcopy referrals. Furthermore, in HR-HPV positive patients with cytology results ≤ASCUS, *PAX1* gene methylation detection significantly decreased colposcopy referral rates (22.29%), thus alleviating patients’ medical burden.

**Conclusion:**

*PAX1* gene methylation detection exhibits strong diagnostic efficiency for cervical lesions and holds significant value in triage diagnosis of non-16/18 HR-HPV positive.

## 1 Introduction

Cervical cancer remains a global issue approximately, severely compromises women’s health (1). It demonstrates one of the highest incidence rates within gynecological neoplasms (2). The pathogenesis of cervical carcinoma involves multifactorial interactions, among which persistent high-risk human papillomavirus (HR-HPV) infection constitutes the predominant etiological determinant (3). Studies confirm that >99% of cervical carcinoma cases exhibit evidence of HR-HPV infection (4). Persistent HR-HPV infection may lead to cervical intraepithelial neoplasia (CIN), which can progress to cervical carcinoma (5). Therefore, early detection and timely intervention are crucial for reducing both the incidence and mortality of cervical cancer (6).

Traditional screening for cervical lesions primarily includes cytology tests (7) and HPV-DNA testing (8). For patients with positive HR-HPV test results, cytology is used as a triage method, and those with results ≥ASC-US should be referred for colposcopy (9). However, cytology cannot reliably distinguish cervical inflammation from more complex conditions, leading to increased colposcopy referral rates (10). Furthermore, most individuals with HR-HPV positive results have transient infections (8) that do not require immediate intervention.Therefore, distinguishing patients who require clinical intervention among HR-HPV-positive individuals remains one of the key challenges in cervical cancer screening.

In recent years, with the continuous advancement of molecular biology technologies, DNA methylation testing has emerged as a promising biomarker detection method (11, 12), demonstrating significant potential in cervical lesion screening and diagnosis (13). DNA methylation is an epigenetic modification involving the covalent addition of a methyl group (-CH₃) to the 5’ carbon of cytosine residues, predominantly occurring at CpG dinucleotides in mammalian genomes (14), Such epigenetic modifications regulate transcriptional activity while preserving the underlying genetic code (15). Carcinogenesis is characterized by promoter CpG island hypermethylation of tumor suppressor genes (TSGs), which recruits DNA methyltransferases (DNMTs) and methyl-CpG-binding domain (MBD) proteins to establish repressive chromatin states, resulting in stable gene silencing (16). Consequently driving oncogenesis (17). The *PAX1*(a paired-box transcription factor) gene serves as a crucial tumor suppressor (16), with aberrant methylation patterns being consistently detected across multiple cancer types (18–22). Studies have demonstrated that *PAX1* methylation levels are closely associated with the severity of cervical lesions (23) and may play a pivotal role in the initiation and progression of cervical cancer (24–27). Therefore, assessing *PAX1* methylation status facilitates early detection of cervical lesions (28) and enables risk stratification based on disease severity.

Our study enrolled cervical exfoliated cell samples from 1,619 female patients to systematically evaluate the diagnostic performance of *PAX1* methylation testing, in conjunction with HPV-DNA testing and cytology, using histopathological assessment as the gold standard. The primary objectives were to investigate the clinical utility of *PAX1* methylation as a cervical lesion screening tool and its potential value in triage strategies.

## 2 Materials and methods

### 2.1 Study population

**Figure 1:**
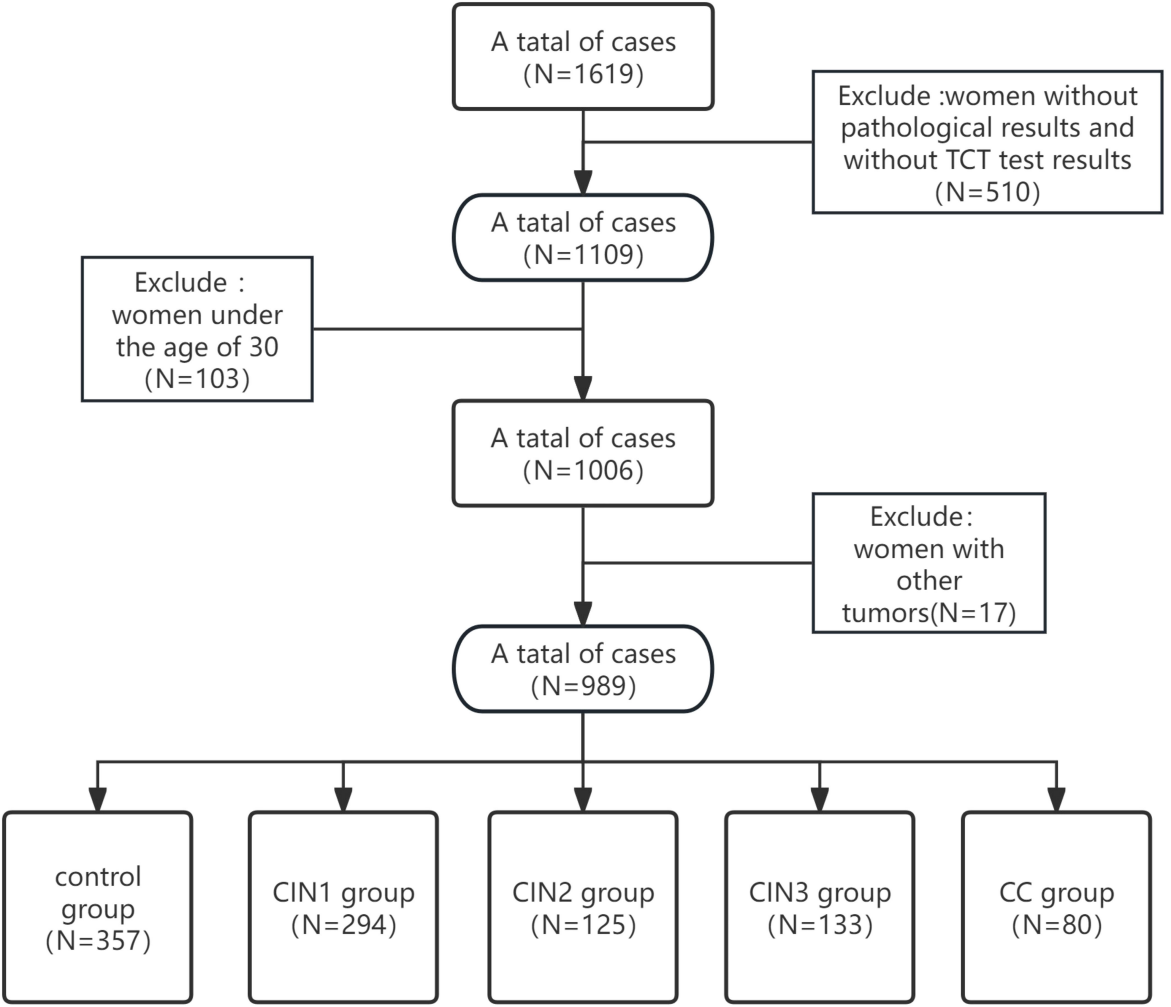
Flowchart of inclusion criteria for current analysis.

A total of 1,619 HPV-positive women were identified from the clinical database of the Affiliated Hospital of Qingdao University (December 2023–March 2025). Colposcopy referrals were triggered by abnormal HPV-DNA or cytology findings.Data were obtained through four diagnostic modalities: colposcopic examination, liquid-based cytology, HPV-DNA genotyping, and quantitative *PAX1* methylation profiling.Cervical tissue specimens were processed and analyzed by board-certified pathologists at the Huangdao Branch of the Affiliated Hospital of Qingdao University, including microscopic examination and immunohistochemical staining.Inclusion Criteria: 1) All patients underwent cervical histopathological confirmation with independent verification by two senior pathologists (holding associate professorship or equivalent qualifications); 2) Diagnostic classification was further confirmed by cytological examination; 3) Healthy controls were defined as either: Routine health examinees at Huangdao Branch, Affiliated Hospital of Qingdao University, orIndividuals with normal cervical histopathology results; 4) Exclusion of concurrent malignancies was confirmed; 5) The age cutoff (≥30 years) aligns with the *PAX1* methylation kit’s (Qingdao Ristad Medical Laboratory Co., Ltd.) intended Use in women ≥30. After applying inclusion criteria and quality control assessments, 630 samples were excluded, leaving 989 eligible samples for final statistical analysis (see **Figure 1**). Histopathological classification was performed by two board-certified pathologists according to the WHO Classification of Tumours (29). Samples were stratified into: control, CIN1, CIN2, CIN3, and cervical cancer (CC) groups. In cases of diagnostic discrepancy (interobserver variability), a consensus diagnosis was achieved through multidisciplinary pathology consultation (“Morbidity & Mortality Conference”), involving collective review of tissue slides and imaging data by additional senior pathologists. This study was approved by the Institutional Review Board of The Affiliated Hospital of Qingdao University (Approval No. QYFYEC2024-306) in accordance with the Declaration of Helsinki principles.

### 2.2 Detection of *PAX1* gene methylation

*PAX1* gene methylation levels were quantitatively analyzed using the Human *PAX1* Methylation Detection Kit (Qingdao Ristad Medical Laboratory Co., Ltd.) on an ABI 7500 Real-Time PCR System (Thermo Fisher Scientific, USA).Quantitative Methylation Analysis: Real-time PCR amplification was conducted using the ABI 7500 Real-Time PCR System (Thermo Fisher Scientific, USA). Fluorescence data were collected and cycle threshold (Ct) values were automatically determined for both the reference gene (ACTB) and target gene (*PAX1*) during the exponential amplification phase. The Ct value represents the number of cycles required for the fluorescence signal to reach a set threshold during PCR, reflecting the initial amount of DNA template. A smaller ΔCt value indicates that the target gene PAX1 has higher amplification efficiency relative to the reference gene ACTB, suggesting a higher methylation level of the PAX1 gene in the sample. This method provides an objective and quantitative reflection of the degree of gene methylation in cell samples, offering reliable evidence for clinical diagnosis.DNA methylation status was assessed by calculating ΔCt values (ΔCt = Ct*PAX1* - CtACTB) while simultaneously examining Curve of expansion (Standard Sigmoid curve). In accordance with Instruction manual of *PAX1* Methylation Detection Kit, samples demonstrating ΔCt values ≤8.31 were classified as *PAX1* methylation-positive, while those with ΔCt values >8.31 were considered methylation-negative.

### 2.3 Detection of HPV‐DNA

Cervical exfoliated cells were collected and preserved in the cell preservation solution at 4°C. All participants were abstained from sexual intercourse, vaginal medication, and douching before 72 h of collection. Human Papillomavirus (HPV) Nucleic Acid Detection and HPV 16/18 Genotyping Kit (Yaneng Biotechnology (Shenzhen) Co., Ltd) was completed using quantitative fluorescence PCR, which could identify 13 types of high-risk HPV (HR-HPV).

### 2.4 Cytology testing

TCT is used for liquid-based cytology examination, sampling requirements and testing procedures are carried out according to the Cancer Prevention Manual issued by IARC in 2005 (30). The diagnoses included: negative for intraepithelial lesion or malignancy (NILM), Atypical squamous cells of undetermined significance(ASCUS), atypical glandular cells (AGC), adenocarcinoma insitu (AIS), atypical squamous cells which cannot exclude HSIL (ASC‐H), High-grade squamous intraepithelial lesion(HSIL), Low-grade squamous intraepithelial lesion(LSIL), Squamous cell carcinoma(SCC). Following the classification presented byPerkins et al. in 2020, we categorized cytology testing result into four groups: NILM, ASCUS, ASC‐H LSIL, and HSIL (31).

### 2.5 Colposcopy testing

Colposcopy is an endoscope used between the naked eye and low-power microscopy. The purpose of colposcopy is to visually inspect the epithelial tissues and blood vessels of the lower genital tract comprehensively, guiding the colposcopist to make a diagnosis while performing biopsies on suspicious lesions. Pre-experiment preparation, experimental procedures, and result interpretation should follow the European consensus statement on expert colposcopy (32).

### 2.6 Statistical analysis

The diagnostic performance of methylation markers was evaluated using the receiver operating characteristic (ROC) curve, with indicators including the area under the curve (AUC), sensitivity, specificity, and their respective 95% confidence intervals (CIs). Inter-group comparisons after normality analysis and homogeneity of variance were conducted using one-way analysis of variance (ANOVA) and *post-hoc* tests(LSD). For categorical data, Pearson χ² tests were used to assess differences between groups. Statistical analyses were performed using IBM SPSS Statistics 26, with statistical significance defined as p<0.05.

## 3 Results

### 3.1 Comparison of general data among groups

**Figure 2:**
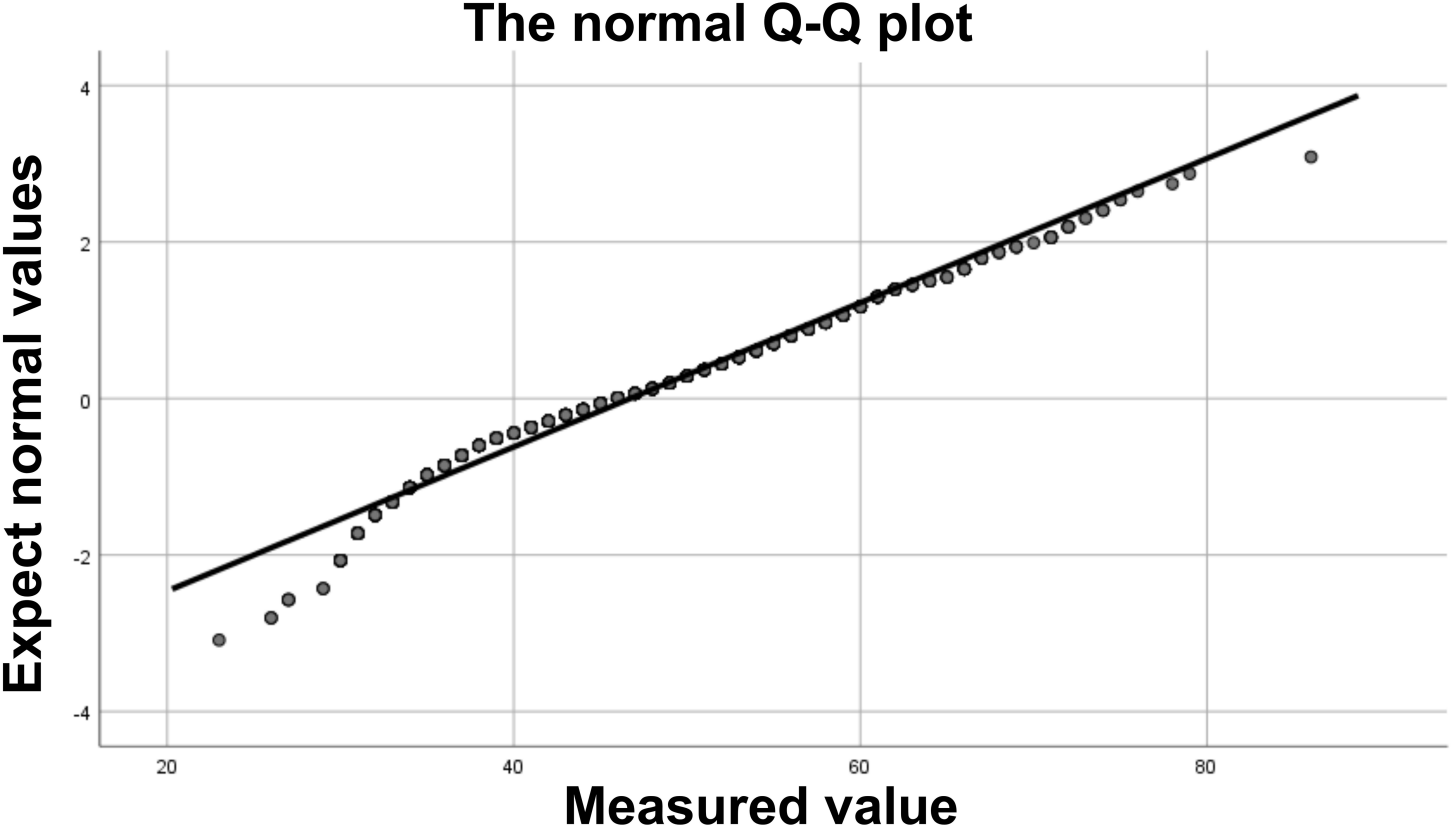
Normal Q-Q plot. The data points form an approximately straight line on the Q-Q plot, indicating that the data approximately follows a normal distribution.

**Table 1** shows the general characteristics of the study participants. A total of 989 female patients were included in this study, with 357 in the control group, 294 in the CIN1 group, 125 in the CIN2 group, 133 in the CIN3 group, and 80 in the CC group. Among these participants, 76 were under 30 years old and 105 were 30 years or older. The age distribution of each group was as follows: control group (26–79 years old, average age 48.6 ± 10.4 years; CIN1 group (23–72 years old, average age 47.7 ± 11.9 years; CIN2 group (26–71 years old, average age 42.8 ± 9.6 years; CIN3 group (30–72 years old, average age 45.2 + 10.2 years; cell carcinoma group (31–86 years old, average age 55.7 + 12.2 years. The age distribution values of each group showed that the absolute value of peakedness was less than 10 and the absolute value of skewness was less than 3. Combined with the normal Q-Q graph (**Figure 2**), the data points showed that the age distribution was close to a straight line, indicating that the age distribution was close to a normal distribution; according to the results of the homogeneity of variance test, the variance of age distribution in each group was homogeneous (p=0.159). One-way ANOVA test showed that the age difference between groups was statistically significant (p <0.001). In terms of methylation positivity rate, the positive rates of the control group, CIN1, CIN2, CIN3 and CC groups were 6.7%, 3.4%, 90.4%, 91.9% and 100% respectively. The one-way ANOVA test showed that there was a significant difference between the groups (p <0.001), and the *post-hoc* test results showed that there was a significant difference in age between the control group and the lesion group (CIN1, CIN2, CIN3 and CC groups) (p <0.001).

**Table 1:** Overview of general data characteristics(N = 989).

Characteristics	Result	N	Proportion (%)	Methylation positivity rate (%)	P‐value
Histopathology					p < 0.001
	Control	357	36.1	6.7	
	Cervical intraepithelial neoplasia (CIN)1	294	29.7	3.4	
	CIN2	125	12.6	90.4	
	CIN3	133	13.4	91.9	
	Cellcarcinoma	80	8.1	100	
High‐risk HPV (hrHPV) test					p < 0.001
	Negative	8	0.8	25	
	16/18 positive	222	22.4	65.4	
	Non‐16/18positive	759	76.7	28.3	
Cytology test					p < 0.001
	Negative for intraepithelial lesion or malignancy	351	35.5	24.8	
	Atypical squamous cells of undetermined significance	266	26.9	24.8	
	Low‐grade squamous intraepithelial lesion	178	18.0	23.6	
	High‐grade squamous intraepithelial lesion/ASC‐H	194	19.6	85.1	

### 3.2 Comparison of methylation positivity rates

**Table 2** shows the methylation positivity rate of samples in each group of high-risk HPV (hrHPV) and cytology detection of samples in each group.Among them, there was a significant difference in the methylation positivity rate between the HPV-negative group, HPV16/18 group, and non-HPV16/18 group (chi-square test P<0.001), with total methylation positivity rates of 25%,65.4%, and 28.3%, respectively. Similarly, there were significant differences in the methylation positivity rate among the NILM, ASCUS, LSIL, and HSIL/ASC-H groups (chi-square test P<0.001), with methylation positivity rates of 24.8%, 24.8%, 23.6%, and 85.1%, respectively.

**Table 2:** Methylation positivity rates in various hrHPV genotypes and cytological test results comparison (N = 989).

Characteristics	Control (n =357)	Cervical intraepithelial neoplasia (CIN1) (n =294)	CIN2 (n =125)	CIN3 (n =133)	Cervical cancer (n =80)	Total	p
hrHPV genotype							p < 0.001
HPV Negative (n =8)	0/4 (0)	0/2 (0)	0	0	2/2 (100%)	2/8 (25%)	
16/18 positive (n =222)	16/50 (32%)	9/4 (22.0%)	17/26 (65.4)	48/52 (92.3%)	53/53 (100%)	143/22 (65.4%)	
Non‐16/18 positive (n = 759)	18/303 (5.9%)	1/25 (0.4%)	96/99 (97.0%)	75/81 (92.6%)	25/25 (100%)	215/75 (28.3%)	
Cytology							p < 0.001
Negative for intraepithelial lesion or malignancy (n = 351)	15/181 (8.3%)	3/98 (3.1%)	32/32 (100%)	37/40 (92.5)	0	87/351 (24.8%)	
Atypical squamous cells of undetermined significance (n = 266)	8/95 (8.4%)	5/113 (4.4%)	34/38 (89.5%)	19/20 (95%)	0	66/266 (24.8%)	
Low‐grade squamous intraepithelial lesion (n =178)	7/66 (10.6%)	2/72 (2.8%)	24/28 (85.7%)	9/12 (75)	0	42/178 (23.6%)	
High‐grade squamous intraepithelial lesion/ ASC‐H (n = 194)	4/15 (26.7%)	0/11 (0)	23/27 (85.2%)	58/61 (95.1%)	80/80 (100%)	165/194 (85.1%)	

### 3.3 Diagnostic performance of methylationdetection for CIN2+, and CIN3+

**Figure 3:**
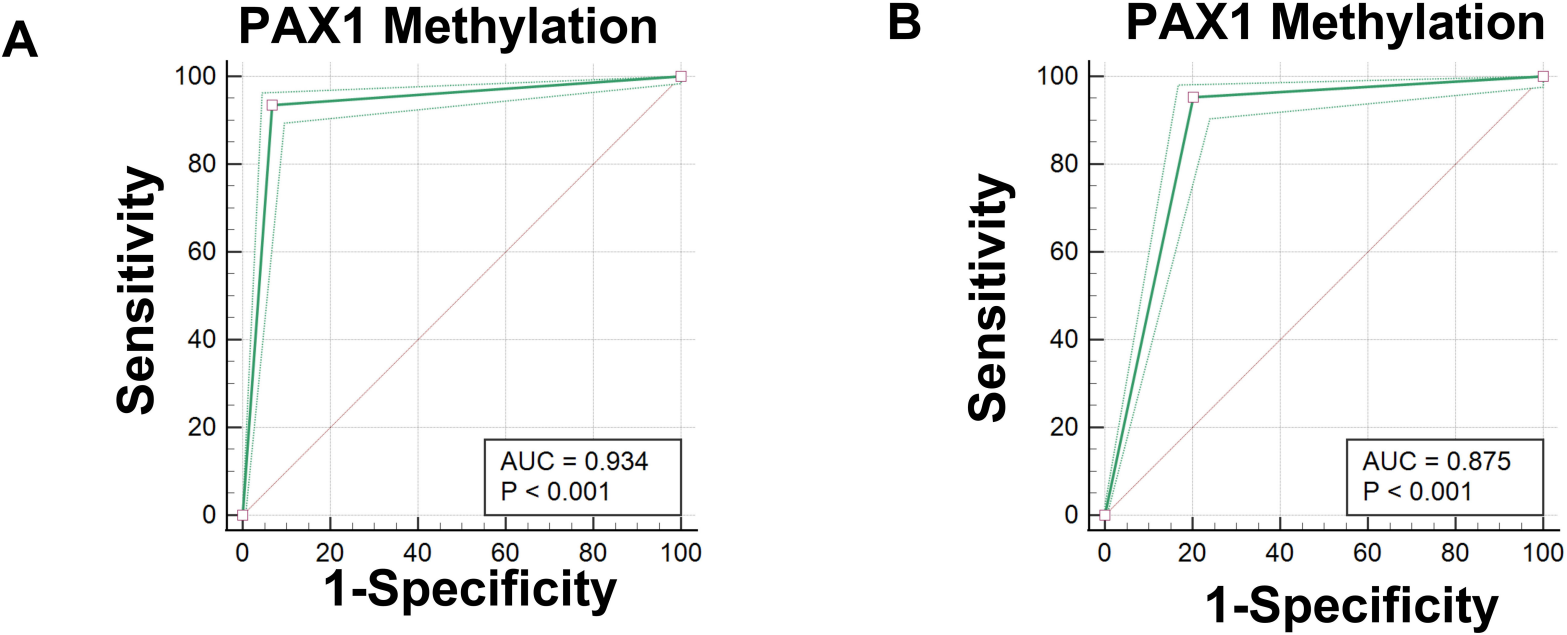
Receiver operating characteristic (ROC) curves for the diagnostic performance of PAX1 methylation in detecting Cervical Intraepithelial Neoplasia (CIN). **(A)** ROC curve for *PAX1* methylation testing with CIN2 as the diagnostic threshold; **(B)** ROC curve for *PAX1* methylation testing with CIN3 as the diagnostic threshold.

**Figure 3**. Diagnostic Performance of *PAX1* Methylation in CIN Detection Receiver operating characteristic (ROC) curve analysis of *PAX1* methylation demonstrated robust diagnostic performance for cervical intraepithelial neoplasia (CIN) detection. Using CIN2 as the diagnostic threshold (**Figure 3A**), *PAX1* methylation exhibited an area under the curve (AUC) of 0.934, with maximum Youden index of 0.86, yielding sensitivity of 93.49% and specificity of 93.24%. When CIN3 was employed as the diagnostic threshold (**Figure 3B**), the analysis revealed an AUC of 0.875, with maximum Youden index of 0.75, corresponding to sensitivity of 95.31% and specificity of 79.77%. These findings indicate that *PAX1* methylation assessment possesses considerable diagnostic value for CIN detection, particularly for CIN2+ lesions.

### 3.4 Comparison of the diagnostic efficiency of cytological and methylation Detection in triage of HPV non‐16/18 positive patients

**Table 3** shows that HPV-DNA was used as an initial screening to identify 222 HPV16/18 positive patients and 759 HPV non-16/18 positive patients. For the latter, it is recommended to further triage through cytology or methylation testing for possible colposcopy referral.

**Table 3:** Comparison diagnostic performance of cytology and methylation testing for triage of HPV non‐16/18 positive cases (N = 759).

Group	Triage method	Sensitivity (%)	Specificity (%)	False negativity (%)	False positivity (%)	Youden’s index
CIN1(251)	Cytology	52.2	64.3	51.2	32.7	0.165
	Methylation	5.3	53.3	5.9	99.6	-0.414
CIN2(99)	Cytology	33.2	87.1	51.2	22.2	0.203
	Methylation	84.2	99.0	5.9	3.0	0.832
CIN3+(106)	Cytology	34.9	86.6	51.2	21.7	0.215
	Methylation	84.7	97.9	5.9	5.7	0.826

Cytology demonstrated high sensitivity (89.2%) but relatively low specificity (64.3%) for CIN1 detection, whereas methylation testing showed lower sensitivity (5.3%) with moderate specificity (53.3%) at this grade. For higher-grade lesions, methylation testing outperformed cytology in both sensitivity and specificity metrics. Specifically, methylation testing achieved sensitivity rates of 84.2% for CIN2 and 84.7% for CIN3+, with corresponding specificity values of 99% and 97.9%, respectively.

Methylation testing yielded high Youden indices for CIN2+ and CIN3+, reaching 0.832 and 0.826 respectively, while exhibiting a negative index (-0.414) for CIN1+. These Youden index values indicate that methylation testing provides excellent sensitivity and specificity for high-grade lesions. In contrast, cytology testing demonstrated consistent performance across all lesion grades, though with both sensitivity and specificity metrics inferior to those achieved by methylation testing for high-grade lesions.

### 3.5 Reduced unnecessary colposcopy referral through methylation detection in HPV‐positive patients with cytology ≤ ASCUS

**Table 4** shows that among the patients who underwent colposcopy, 617 samples had cytology test results. Among the <ASCUS samples, 348 were positive for hr-HPV; among the ASCUS samples, 264 were positive for hr-HPV. In the <ASCUS samples, 70 cases were CIN2+ methylation positive; in the ASCUS samples, 87 cases were CIN2+ methylation positive. Overall, 157 cases were methylation positive, resulting in a colposcopy referral rate of 22.29% (157 out of 617 cases).

**Table 4:** Decrease in unnecessary colposcopy referrals through methylation detection in HPV‐positive patients with cytology ≤ atypical squamous cells of undetermined significance (ASCUS) (N = 617).

Cytology result	hrHPV‐positive	Methylation result	Control	CIN1	CIN2	CIN3+	Total
<ASCUS (n = 351)	348/351 (99.1%)	Positive	15 (8.3%)	3 (3.1%)	33 (100%)	37 (92.5%)	/
		Negative	166 (91.7%)	95 (96.9%)	0	3 (7.5%)	264
ASCUS (n = 266)	264/266 (99.2%)	Positive	8 (8.4%)	5 (4.4%)	34 (89.5%)	53 (91.4%)	/
		Negative	87 (91.6%)	108 (95.6%)	4 (10.5%)	5 (8.6%)	204

## 4 Discussion

This study retrospectively analyzed the data of 989 clinical samples and deeply explored the application value of *PAX1* gene methylation detection in cervical lesion screening and its potential advantages in triage.Research findings indicate that *PAX1* gene methylation testing exhibits superior diagnostic efficacy in identifying high-grade cervical lesions (CIN2+ and CIN3+), particularly among patients positive for non-16/18 high-risk HPV genotypes, when compared to conventional cytological assessment (33). Methylation testing demonstrates superior efficacy in reducing unnecessary colposcopy referrals, providing novel insights and scientific evidence for optimizing cervical cancer screening strategies.

### 4.1 Diagnostic Performance of PAX1 Gene Methylation Detection

In this study, *PAX1* gene methylation testing demonstrated significantly superior diagnostic performance compared to cytological examination for detecting CIN2+ and CIN3+. When using CIN2 as the diagnostic threshold, *PAX1* methylation testing achieved an AUC value of 0.934, with a sensitivity of 93.49% and specificity of 93.24%. When using CIN3 as the diagnostic threshold, the AUC value was 0.875, with a sensitivity of 95.31% and specificity of 79.77%. The results demonstrate that *PAX1* gene methylation testing exhibits high accuracy in identifying high-grade cervical lesions, effectively avoiding missed diagnoses and misdiagnoses. In contrast, while cytological examination shows relatively high sensitivity (89.2%) for CIN1, its specificity is lower (64.3%), and its diagnostic performance for CIN2 and CIN3+ stages is significantly inferior to methylation testing. This discrepancy may be attributed to cytology’s primary reliance on cellular morphological changes, which are often not sufficiently pronounced in low-grade lesions and can be influenced by subjective factors.Studies have shown that *PAX1* gene methylation testing demonstrates significantly superior diagnostic performance compared to cytological examination for detecting CIN2+ and CIN3+ cervical lesions (32). As a molecular biological detection method, *PAX1* gene methylation testing can directly reflect the genetic status within cells, offering higher sensitivity and specificity for early identification of lesions and assessment of their severity.

### 4.2 The Triage Value of Methylation Testing in HR-HPV Non-16/18 Positive Patients

Studies have found that methylation testing demonstrates high triage efficacy in patients who are positive for high-risk HPV types other than 16/18 (34). Our study results show that methylation testing demonstrates a sensitivity of 84.2% and 84.7% for CIN2 and CIN3+ respectively, with specificity of 99% and 97.9% respectively, which is significantly higher than cytology testing. This indicates that in HR-HPV non-16/18 positive patients, *PAX1* gene methylation testing can more accurately identify patients requiring further intervention, reducing unnecessary colposcopy referrals.Although cytology testing has high sensitivity in CIN1 stage, it has limitations in identifying high-grade lesions (35), which can lead to over-referral. Methylation testing, by precisely identifying the degree of lesions, can effectively avoid unnecessary medical interventions and improve the efficiency of medical resource utilization.

### 4.3 Reduction of unnecessary colposcopy referrals

This study also explored the potential value of *PAX1* gene methylation testing in reducing unnecessary colposcopy referrals. Among patients who were HR-HPV positive with cytology results ≤ASCUS, the positivity rate of methylation testing was significantly lower than that of cytology testing, indicating that methylation testing can effectively screen out patients who truly need colposcopy examination.The study results showed that among 617 patients who underwent colposcopy, the positive rate of methylation testing was 22.29%, which was significantly lower than the positive rate of cytology testing. This indicates that *PAX1* gene methylation testing can significantly reduce unnecessary colposcopy referrals, alleviate patients’ medical burden, and avoid potential risks associated with excessive examinations.

### 4.4 Limitations and Prospects

The discussion of sample limitations aligns with standard practices in medical research methodology. For *PAX1* methylation studies specifically, multi-center trials with diverse populations(different regions and ethnic groups were included) are indeed needed to establish broader clinical utility, as suggested in the literature on cancer biomarkers and molecular diagnostics. Secondly, despite *PAX1* gene methylation testing demonstrating considerable efficacy in diagnostic performance, its implementation faces economic constraints that may impede widespread adoption in primary healthcare settings. Future investigations should prioritize methodological optimization to enhance cost-effectiveness and accessibility across diverse healthcare environments. In addition, this study mainly focused on the application of PAX1 gene methylation detection in cervical lesion screening, without in-depth exploration of its potential value in cervical cancer treatment monitoring and prognosis evaluation. Future research could further expand the scope to explore the application value of PAX1 gene or other gene methylation detection in the comprehensive management of cervical cancer.

## 5 Conclusion

In conclusion, PAX1 gene methylation testing demonstrates high diagnostic efficacy for cervical lesions and holds significant clinical value in triage assessment of HR-HPV non-16/18-positive cases.

## Data Availability

The raw data supporting the conclusions of this article will be made available by the authors, without undue reservation.
